# Speciation with gene flow between two Neotropical sympatric species (*Pitcairnia* spp.: Bromeliaceae)

**DOI:** 10.1002/ece3.8834

**Published:** 2022-04-29

**Authors:** Marília Manuppella Tavares, Milene Ferro, Bárbara Simões Santos Leal, Clarisse Palma‐Silva

**Affiliations:** ^1^ Departamento de Biologia Vegetal Instituto de Biologia Universidade Estadual de Campinas Campinas Brazil; ^2^ Departamento de Biologia Geral e Aplicada Universidade Estadual Paulista Rio Claro Brazil

**Keywords:** Bromeliaceae, divergent selection, hybridization, NGS, reproductive isolation, speciation with gene flow

## Abstract

The study of mechanisms that generate new species is considered fundamental for broad areas of ecology and evolution. Speciation is a continuous process in which reproductive isolation is established, and it is of fundamental importance to understand the origins of the adaptations that contribute to this process. Hybrid zones are considered natural laboratories for the study of speciation and represent ideal systems for such studies. Here, we investigated genomic differentiation between hybridizing Neotropical species *Pitcairnia staminea* (G. Lodd.) and *P. albiflos* (Herb.). Using thousands of SNPs genotyped through RAD‐seq, we estimate effective population sizes, interspecific gene flow, as well as time of divergence between these two sister species and identify candidate genomic regions for positive selection that may be related to reproductive isolation. We selected different scenarios of speciation and tested them by using approximate Bayesian computation (ABC); we found evidence of divergence with gradual reduction in gene flow between these species over time, compatible with the hypothesis of speciation with gene flow between these *Pitcairnia* species. The parameter estimates obtained through ABC suggested that the effective population size of *P. albiflos* was around three times larger than that of *P. staminea*. Our divergence date estimates showed that these two species diverged during the Pliocene (4.7 Mya; CI = 1.3–8.5 Mya), which has likely allowed this species to accumulate genome‐wide differences. We also detected a total of 17 of 4165 loci which showed signatures of selection with high genetic differentiation (*F*_ST_ > 0.85), 12 of these loci were annotated in de novo assembled transcriptomes of both species, and 4 candidate genes were identified to be putatively involved in reproductive isolation. These four candidate genes were previously associated with the function of pollen development, pollen tube germination and orientation, abiotic stress, and flower scent in plants, suggesting an interplay between pre‐ and postpollination barriers in the evolution of reproductive isolation between such species.

## Graphical abstract

Graphic representation of the simulated scenarios of divergence between *Pitcairnia albiflos* and *P. staminea* tested using approximate Bayesian computation (ABC) framework. By contrasting different scenarios of speciation using ABC, we found evidence of divergence with gradual reduction in gene flow between these species over time, compatible with the hypothesis of speciation with gene flow between these Neotropical plant species.

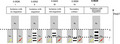

## 1 INTRODUCTION

The process of speciation can be characterized by the evolution of barriers to gene flow, which includes ecological isolation, intrinsic genetic incompatibilities, and selection against hybrids in nature (Seehausen et al., 2014). When it is driven by divergent selection, prezygotic and extrinsic postzygotic barriers are predicted to evolve first, and interactions between them may reduce gene flow between populations, and intrinsic postzygotic barriers may evolve later (Seehausen et al., 2014; Stankowski & Ravinet, 2021). Also, the initial conditions (sympatry or geographic isolation) can affect how differentiation occurs (Feder et al., 2012; Flaxman et al., 2014; Nosil et al., 2017). Although allopatric speciation requires geographical isolation and time, allowing populations to diverge by processes such as ecological selection and/or drift due the lack of gene flow (Turelli et al., 2001), sympatric or parapatric speciation depends on the balance of divergent selection and gene flow, being strongly facilitated if traits under divergent selection also contribute to assortative mating (Dieckmann & Doebeli, 1999; Kautt et al., 2020; Niemiller et al., 2008; Papadopulos et al., 2011; Rice & Hostert, 1993; Turelli et al., 2001).

Currently, there are several lines of evidence for speciation with gene flow in plants (e.g., Muniz et al., 2022; Papadopulos et al., 2011, 2019; Roberts & Roalson, 2018) as well as in animals (e.g., Camurugi et al., 2021; Kautt et al., 2016, 2020; Martin et al., 2013; Niemiller et al., 2008; Oliveira et al., 2015; Roux et al., 2016). One prediction of this model is that the level of divergence should be heterogeneous across the genome, given that alleles at some loci are likely to be shared between incipient species, while selection maintains divergence at other loci (Feder et al., 2012; Nosil et al., 2009; Riesch et al., 2017; Seehausen et al., 2014; Turner et al., 2005; Wu, 2001). Moreover, divergence can also be driven by selection on several independent genomic regions (Michel et al., 2010; Nosil et al., 2017, 2021). Barriers to gene flow can be facilitated by numerous genes of small effect distributed widely in the genome, or by few genes with large effect on reproductive isolation (Sedeek et al., 2014). Thus, the accumulation of genomic divergence between incipient species occurs across the speciation continuum, usually starting by an initial phase of divergent selection on few loci (Via & West, 2008). In theory, sudden speciation is also compatible with evolution in small steps because linkage disequilibrium and divergent selection can reach threshold levels and enhance each other in a positive feedback loop, speeding processes involving small changes up at genome wide (Flaxman et al., 2014; Nosil et al., 2017, 2021). Hence, advances in next‐generation sequencing (NGS) are enabling researchers to address these questions through comparative studies of genome‐wide patterns of differentiation between populations at varying stages along the speciation continuum (Feder et al., 2012; Martin et al., 2013; Nosil & Feder, 2012).

Hybridization may occur under multiple geographical scenarios of speciation, and is more commonly found in parapatric and sympatric processes (Abbott et al., 2013; Seehausen, 2004). Several animal and plant species hybridize regularly and closely related species tend to hybridize more often (Abbott et al., 2013; Mallet, 2005; Seehausen, 2004). While it can result in reduction or loss of differentiation in some cases (e.g., Balao et al., 2015; Lepais et al., 2009), hybridization may represent an important source of adaptive genetic variation through introgression of selectively favored alleles from one population into another. Interspecific gene flow may increase the genetic diversity of introgressed taxa and contribute to speciation process and may act as a source of variation for rapid adaptive radiations (Abbott et al., 2013; Gardner & Vose, 2005; Lexer et al., 2016; Loiseau et al., 2021; Mallet, 2007; Palma‐Silva et al., 2016; Runemark et al., 2019; Seehausen, 2004). In this context, Bromeliaceae family is one of the best characterized and studied example of adaptive radiation in Neotropics (Barbará et al., 2008; Givnish et al., 2011, 2014, 2015; Palma‐Silva, Leal, et al., 2016). Interspecific gene flow has been largely reported in this plant family (Gardner, 1984; Goetze et al., 2017; Gonçalves & de Azevêdo‐Gonçalves, 2009; Mota et al., 2019; Mota et al., 2020; Neri et al., 2017; Palma‐Silva et al., 2011; Palma‐Silva et al., 2015; Schulte et al., 2010; Wendt et al., 2001; Zanella et al., 2016) and both ancient and contemporary hybridization are considered to be important to the diversification of this Neotropical radiation (i.e., Goetze et al., 2017; Loiseau et al., 2021; Neri et al., 2017; Mota et al., 2019; Mota et al., 2020, and see review of Palma‐Silva, Leal, et al., 2016). A growing body of studies based on molecular markers evaluating hybridization and interspecific gene flow between species of Bromeliaceae have shown that, despite hybridization, the interplay between pre‐ and postzygotic reproductive isolation barriers is important in maintaining species integrity (e.g., Lexer et al., 2016; Matallana et al., 2010; Mota et al., 2019, 2020; Neri et al., 2017; Palma‐Silva et al., 2011, 2015; Schulte et al., 2010; Souza et al., 2017; Wagner et al., 2015; Zanella et al., 2016). Therefore, studies of interspecific gene flow based on a large dataset of high‐throughput sequencing data in bromeliads could help us to understand how the evolution of reproductive isolation barriers promotes speciation with gene flow in this group (Palma‐Silva, Leal, et al., 2016).

*Pitcairnia staminea* and *P. albiflos* are two closely related diploid species, narrowly endemic to inselbergs in Rio de Janeiro, Brazil, where they occur either allopatrically or sympatrically at local scale (Palma‐Silva et al., 2011, 2015; Wendt et al., 2001). Inselbergs are isolated rock outcrops that rise abruptly above the surrounding plains, conferring spatial and ecological isolation as a barrier against dispersal and migration (Porembski, 2007), which make them good models for studying ecological and evolutionary diversification (Barthlott & Porembski, 2000). These species are morphologically well defined, while *P. albiflos* has white flowers, *P. staminea* has red flowers. They also diverge in their habitat preferences, with the former occurring in open and sunny patches, and the later in patches often shaded by scrubs and trees (Wendt et al., 2001). The current existence of hybrids and interspecific gene flow between sympatric populations of *P. staminea* and *P. albiflos* has been confirmed notwithstanding strong reproductive barriers previously described, i.e., divergent floral traits and phenology, shift in mating system, and genetic incompatibilities (Palma‐Silva et al., 2011, 2015; Wendt et al., 2001). In this study, we aim to deepen the investigation of the genetic bases of speciation between these species addressing the following questions: (1) Does the speciation involve genetically localized regions or genome‐wide changes? (2) Which genomic regions are possibly associated with reproductive isolation between these two species? (3) Has the divergence between species occurred with migration, evidencing a case of speciation with gene flow? We used single nucleotide polymorphisms (SNP) data derived from RAD sequencing to investigate the genome‐wide pattern of differentiation between them and to identify which genomic regions are possibly related to reproductive barriers. Additionally, we applied the approximate Bayesian computation (ABC) framework to compare distinct speciation scenarios, as well as estimate gene flow across the divergence process and the divergence time.

## 2 MATERIALS AND METHODS

### 2.1 Study system and sampling

We selected 12 specimens of each of the two species *Pitcairnia albiflos* and *P. staminea* (total 24 individuals) from the most well–studied hybrid zone between them, located in an inselberg named “Pão de Açúcar,” in the Serra do Mar Mountain located in Rio de Janeiro city, Southeast Brazil. All specimens were previously analyzed with nuclear microsatellites (Palma‐Silva et al., 2011; data not shown) in order to classify them and avoid sampling hybrid and introgressed individuals that might bias our results of gene flow between species. Previous studies have confirmed the existence of historical and contemporary interspecific gene flow between these two species despite strong pre‐ and postzygotic reproductive barriers (Palma‐Silva et al., 2011, 2015; Wendt et al., 2001). In fact, divergent floral traits and mating systems suggest strong reproductive isolation between species. *Pitcairnia albiflos* has nocturnal anthesis, and white and scented flowers visited by nocturnal animals such as bats and hawk moths, while *Pitcairnia staminea* has red and scentless diurnal flowers and is pollinated by butterflies, although both species are visited by trigonid bees all over the day (Wendt et al., 2001). In addition, *P. albiflos* is dependent on pollinators for fertilization while *P. staminea* is capable of higher levels of self‐fertilization in the absence of pollinators (Wendt et al., 2001). *Pitcairnia staminea* also presented higher selfing rates and reduced herkogamy in sympatric than in allopatric populations, suggesting reinforcement of assortative mating may be important in the evolution of reproductive isolation (Palma‐Silva et al., 2015). In addition, our previous studies have shown that Bateson–Dobzhansky–Muller (BDM) incompatibilities may contribute as postzygotic reproductive barrier between such species, as suggested by the asymmetric introgression rates of some nuclear loci (Palma‐Silva et al., 2011).

### 2.2 RAD‐Seq library preparation, de novo assembly, and SNP calling

Genomic DNA was extracted from silica gel dried leaves of each sampled individual using the Qiagen DNA plant mini kit (Qiagen, Finland). Library preparation and sequencing of RAD markers from genomic DNA of the 12 samples from each species was performed by Floragenex Inc. (Eugene, Oregon) using the restriction enzyme *SbfI* and sample‐specific barcodes. The amplification and single‐ended sequencing (150 pb) were performed on a single lane of Illumina HiSeq 2000 platform.

**FIGURE 1:**
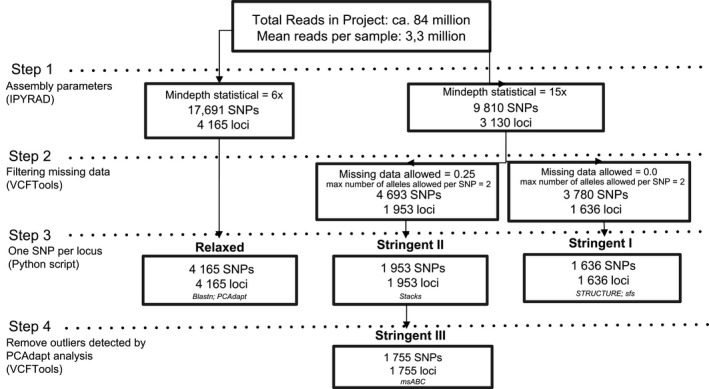
Summary of datasets obtained from *de novo* assembly of 24 samples of *Pitcairnia albiflos* and *Pitcairnia staminea*. Step 1: Assembly: Mindepth statistical: the minimum depth at which statistical base calls will be made during consensus base calling. Step 2: filtering of missing data and biallelic SNPs. Step 3: pick up one random SNP per locus. Step 4: removal of outliers loci detected by PCAdapt analysis. The software used are in parentheses, posterior analyses of each dataset are in italic and the names of each dataset are in bold

The quality of raw reads was checked using FastQC v.0.11.3 (Andrews, 2014) and alignments were exported as bam files, sorted, and indexed using SAMtools version 0.1.16 (Li et al., 2009). The GC content was checked using BBMap v.35.85 (Bushnell, 2014) (Table S1). We then used iPyrad v.0.7.30 (Eaton, 2014) on the server of the Center for Scientific Computing (NCC/GridUNESP) of the São Paulo State University (UNESP) to filter low‐quality base calls (Q < 20), trim adaptors, and perform de novo assembly using the VSEARCH tool (Rognes et al., 2016). Because it is well known that parameter changes affect the joint estimation of sequencing error rate and sample heterozygosity (Eaton, 2014), we clustered the sequences into loci using two different sets of parameters, named “stringent” and “relaxed.” The first was used for analyses that require more stringent data and the second for identification of candidate loci for local adaptation analysis (Figure 1). For both datasets, the default settings of parameter files generated by iPyrad were used except for the two parameters that affect the minimum coverage depth (i.e., mindepth_statistical and mindepth_majrule), which was increased from 6 times in the relaxed dataset to 15 times in the stringent dataset. As analyses require non‐linked single nucleotide polymorphisms (SNPs), we used a custom Python script to pick up one random SNP per locus. We then employed VCFTOOLS v.0.1.17 (Danecek et al., 2011) to retrieve only biallelic loci and filter according to two different amounts of missing data in the stringent assembly: allowing a maximum of 25% missing data per locus (stringent II), which was used for diversity analysis, and no missing data (stringent I) used for structure analysis (Figure 1). Additionally, we performed an outlier test using PCAdapt (Luu et al., 2018) in the stringent II dataset. To eliminate spurious outliers, we used the Bonferroni correction method with a FDR of 0.01. We filtered for outliers detected (total of 198 outlier loci) to produce a dataset with only neutral loci (stringent III) that we used for ABC analysis (Figure 1).

### 2.3 Preprocessing, de novo assemblies, and quality control of transcriptomes

We obtained raw RNA‐sequencing data from *Pitcairnia albiflos* and *P. staminea* previously generated by Palma‐Silva et al. (2016) deposited in the GenBank SRA database (BioProject number PRJNA297023). Here, we performed de novo transcriptome assembly using Trinity (version 2.0.6, Grabherr et al., 2011) with default parameters, assembling together reads from leaves and flowers tissues per each species (*P. albiflos* and *P. staminea*) (see Table S2).

We evaluated the transcriptomes completeness using Benchmarking Universal Single‐Copy Orthologs (BUSCO) v4.0.4 (Seppey et al., 2019; Simão et al., 2015). The transcriptomes for both species were compared with a predefined set of Eukaryota single‐copy orthologs from the OrthoDB v10 database, considering a cut‐off of 1E‐3. Contigs were categorized as “complete, single copy,” “complete, duplicated copy,” “fragmented,” or “missing,” depending on the length of the aligned sequence.

For each species transcriptome assembled, coding protein sequences (CDS) were found using OrfPredictor v3.0 (Min et al., 2005) program. To identify orthologous gene clusters between *P. albiflos* and *P. staminea*, we used the web platform OrthoVenn (Wang et al., 2015). This program uses a modified version of OrthoMCL (Li, 2003) to find ortholog groups. We used an inflation value of 1.5 and E‐value cutoff of 1e−2 for the generation of orthologous clusters. OrthoVenn performs all‐against‐all protein (BLASTP) alignment and identifies putative orthologous and paralog relationships using a Markov Clustering Algorithm—MCL (Enright, 2002). Only orthologous groups with single‐copy genes (only one sequence for each species) were retained for detection of natural selection signatures (Ka/Ks ratio tests—see details below).

### 2.4 Genomic divergence and genetic diversity

To estimate the genomic admixture and ancestry between the two species, we performed a model‐based clustering method using STRUCTURE version 2.3.4 (Pritchard et al., 2000) using the stringent I dataset (Figure 1) obtained through RAD sequencing. The analysis was carried out under the admixture model assuming independent allele frequencies and using a burn‐in period of 50,000 and 250,000 MCMC iterations. The method of Evanno et al. (2005) implemented in STRUCTURE HARVESTER v0.6.97 (Earl & vonHoldt, 2012) was further employed to determine the most likely number of clusters (K) present in the data. Additionally, to describe the distribution of sample allele frequencies, we constructed a site frequency spectrum (SFS) using the stringent I dataset and a Python script available on github (https://github.com/isaacovercast/easySFS). This approach shows the proportion of SNPs with alternative and fixed alleles.

Using the dataset stringent II, we calculated diversity indices, such as the number of private alleles, observed heterozygosity (HO), expected heterozygosity (HE), and inbreeding coefficient (*F*_IS_) for each species and also the calculated *F*_ST_ between species using the program populations in Stacks (Catchen et al., 2011).

### 2.5 Identification of candidate loci potentially under selection

To identify SNPs potentially under selection, we used the relaxed dataset (4165 loci) and a differentiation‐based method in the software PCAdapt (Luu et al., 2016). This method infers population structure with latent factors and assumes that markers excessively related to population structure are candidates for local adaptation, identifying outlier SNPs based on the multidimensional Mahalanobis distance (Luu et al., 2018). To eliminate spurious outliers, we used the Bonferroni correction method with a FDR of 0.01. For this analysis, the initial number of principal components (K) was set to 10 and we used the graphical approach based on the screen plot to choose the number of K.

As species showed high differentiation spread across the genome (see results below), we performed an alternative approach to outlier loci in PCAdapt to identify genomic regions under selection in this study. Therefore, we integrated information on the synonymous and non‐synonymous mutations ratio (Ka/Ks) from de novo assembled and annotated transcriptomes and the genetic differentiation index of corresponding RAD sequences of relaxed dataset.

We used the assembled transcriptomes per species to select orthologous genes that showed to be under positive selection by the Ka/Ks analysis. To obtain genes under positive selection, the protein‐coding sequence orthologs alignment for each single‐copy orthologous group was implemented with Parallel Alignment and Translation (ParaAT) v1.0 (Zhang et al., 2012) with the multiple‐sequence alignment program specified as MAFFT. Non‐synonymous sites (Ka), synonymous sites (Ks), and the ratio Ka/Ks were estimated with KaKs Calculator v2.0 (Zhang et al., 2006) with the model averaging (MA) method. Ka/Ks > 1 indicates positive selection. This analysis quantifies selection pressures by comparing the rate of substitutions at silent sites to the rate of substitutions at non‐silent sites (Kryazhimskiy & Plotkin, 2008).

Functional annotation of positively selected genes was done using the OmicsBox (BioBam, Valencia, Spain) program that is an updated version of Blast2GO (Götz et al., 2008). This tool describes the sequences using BLAST (Altschul et al., 1990) against databases specified by the user, searches by protein domains using the InterProScan program, and performs the gene ontology (GO) terms mapping. We used the Blastx‐fast program against the NR database based on a minimum E‐value of 1e‐03. To search for protein domains, we used all InterProScan databases available. REVIGO Web server (Supek et al., 2011, http://revigo.irb.hr) was used to summarize and visualize GO terms based on a clustering algorithm that relies on semantic similarity measures. Terms were clustered at a specified similarity cutoff of 0.9 (a “large” set) considering SimRel semantic similarity measure. The detailed biological processes of representative terms are listed in Appendix S1.

To identify SNPs in transtrips that showed to be under positive selection, we mapped the RAD sequences of the relaxed dataset (4165 loci) against the dataset of annotated contigs of the assembled transcriptomes using Blastn algorithm. To further evaluate if the RAD selected loci might be under selection, we also used the genetic differentiation index (*F*_ST_) between species of each locus, as calculated by VCFtools v.0.1.17 (see Appendix S2).

### 2.6 Approximate Bayesian computation

**FIGURE 2:**
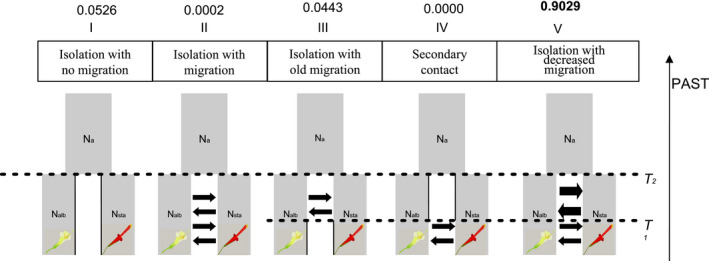
Graphic representation of the simulated scenarios of divergence between *Pitcairnia albiflos* and *Pitcairnia staminea* tested using Approximate Bayesian computation (ABC) framework, with estimated posterior probabilities based on 5000 retained simulations using data transformed by Principal Component Analysis and the neural network algorithm. T2: divergence time; T1: time for cessation of gene flow in old migration model, initiation of migration in the secondary contact model and change of migration rates in the isolation with decreased migration model

We used an ABC framework with the stringent III dataset to test different scenarios of divergence between *P. albiflos* and *P. staminea* and to estimate the intensity of gene flow during the speciation process. All models included a divergence event in an ancestral population that produced two descendant lineages. We generated 100,000 simulated datasets using the program msABC (Pavlidis et al., 2010) under five distinct scenarios: (I) isolation with no migration; (II) isolation with migration; (III) isolation with old migration; (IV) secondary contact; and (V) isolation with decreased migration (Figure 2). The command lines for each model are listed in Table S3.

Priors were initially chosen to represent parameters values that are commonly encountered in empirical studies. Values for the mutation rate were fixed at 10⁻⁹ (Ossowski et al., 2010), recombination rate was set to 0, and we considered a generation time of 5 years for these species (personal observation). As locus vary in size, parameter values of theta (θ) were calculated for each locus using the mutation rate, the length in pairs of bases of each loci, and the ancestral population size (*N* = 10,000). Parameter values of divergence time (T_2_), time of old migrations (T_1_), migration rates, and population sizes were drawn from prior distributions (Table 2).

We initially ran our simulations to produce all the available summary statistics that msABC calculates for the simulated and observed data. Then, using the R‐package “abc” version 2.1 (Csilléry et al., 2012), we selected the following five summary statistics based on the goodness of fit of each statistic to our observed data (Figure S3) and also on the absence of redundancy among them: Tajima's D for each species, *F*_ST_, proportion of shared alleles, and proportion of fixed alleles. Additionally, we transformed the five summary statistics of simulated data by a principal component analysis (PCA). Following completion of the simulation and summary statistics calculation steps, we used the R‐package “abc” for model selection with both datasets (original and PCA transformed) based on the neural network method on 5% retained simulations.

We also performed a parameter estimation step for all parameters (old and recent migration rates, time of old migrations, divergence time, and effective population size of both populations) based on 5000 posterior samples simulated under the best model (V‐isolation with decreased migration) using the neural networks approach.

## 3 RESULTS

### 3.1 RAD‐Seq sequencing and assembled data

We obtained a total of 84,007,380 Illumina reads (min. 2,138,001 and max. 4,004,967 per sample) (Table S1). A total of 17,691 SNPs and 4165 loci were obtained with the relaxed set of parameters and 9810 SNPs and 3130 loci with the stringent assembly (Figure 1). Two datasets were further obtained from the stringent assembly with varying amounts of missing data: 4693 SNPs and 1953 loci (25% of missing data) and 3780 SNPs and 1636 loci (without missing data). After filtering for one random SNP per locus, the two final datasets had the number of SNPs equal to the number of loci (Stringent I: 1636 loci and SNPs and Stringent II: 1953 loci and SNPs). Also, we performed an outlier analysis using PCAdapt on the Stringent II dataset, which resulted in a fourth dataset (Stringent III: 1755 loci and SNPs) (Figure 1).

### 3.2 Transcriptomes assembly and orthologous genes

We obtained 215,000,000 and 193,296,338 preprocessed reads per *P. albiflos* and *P. staminea*, respectively. In the new de novo assembly, we generated 147,097 and 152,819 transcripts for each species, respectively (Table S2). The BUSCO analysis for the Eukaryota database revealed a high number of complete genes for each assembled transcriptome, i.e., 96.4% for *P. albiflos and* 96.3% for *P. staminea*. These results also confirmed the low fragmentation in these assemblies, with fragmented and missing genes corresponding to only 3.6% for *P. albiflos* and 3.7% for *P. staminea*.

OrfPredictor indicated that 145,788 from the *P. albiflos* transcriptome and 151,643 from the *P. staminea* transcriptome are coding sequences (CDS) (Table S2). The total of CDS for each species was further used as input to run OrthoVenn. We identified a total of 47,636 shared orthologs between *P. albiflos* and *P. staminea* species and used the 33,909 single‐copy genes to perform the Ka/Ks ratio test.

### 3.3 Genomic divergence and genetic diversity

Genomic admixture analysis with STRUCTURE identified K = 2 as the most likely number of genetic clusters suggesting minimal evidence of introgressed ancestry between the two species. Twelve sampled individuals were assigned to each group that corresponded to the *P. staminea* and *P. albiflos* sampled individuals (Figure S1). The SFS also indicated the presence of two distinct groups, showing remarkable differences in allele frequencies between *P. staminea* and *P. albiflos* populations (Figure S1). Additionally, we estimated high and significant average genetic differentiation across loci (*F*_ST_ = 0.331). When removing the outliers loci, the average *F*_ST_ across loci remained high (=0.249).

From the 1953 loci per population (stringent II dataset), 877 private alleles were observed in *P. albiflos* and 641 in *P. staminea*. Population‐level inbreeding coefficients were lower in *P. albiflos* than in *P. staminea* (*F*_IS_ = 0.164 and 0.202, respectively) as well as the difference between observed and expected heterozygosities (*P. albiflos*: HE = 0.137 and HO = 0.107; *P. sataminea*: HE = 0.105 and HO = 0.059).

### 3.4 Identification of loci under selection

PCAdapt detected 442 outlier loci on the Relaxed dataset. The results indicated that of the 4271 contigs that showed to be under positive selection by the Ka/Ks analysis, 70 had a significant hit with RAD tags from Relaxed dataset (4165 loci) (see Appendix S3). From those 70 RAD tags with a candidate function, we selected the sequences with *F*_ST_ values higher than 0.85 between species, resulting in a total of 17 loci, of which 12 could be annotated using blastn against the reference transcriptome of the two species (Table 1). Of these, four are associated with genes with putative reproductive function in other plant species: major pollen allergen, potentially implicated in pollen germination in olive (Barral et al., 2020); jasmonic acid‐amido synthetase, regulate pollen maturation in *Arabidopsis* (Turner et al., 2002); inositol phosphorylceramide glucuronosyltransferase, the only transcript also detected as outlier loci in the PCAdapt, putative roles in plant defense, growth, and reproduction in *Arabidopsis* (Tartaglio et al., 2017); and UDP‐galactose/UDP‐glucose transporter, essential for pollen development in *Arabidopsis thaliana* (Reyes et al., 2010). Detected functions are shown in Figure S2 summarized by REVIGO using GO terms obtained for the positive selected genes.

**TABLE 1:** Annotated putative sequences under positive selection

Transcript name	RNA‐seq			RAD‐seq
	Blast annotated function	Trascriptome length (bp)	Ka/Ks values (>1)	*F*_ST_
TRINITY_DN10315_c1_g1_i1_alb	Pentatricopeptide repeat‐containing protein At5g64320	3200	50.000	1.000
**TRINITY_DN21171_c0_g1_i1_alb**	**Major pollen allergen Ole e 10‐like**	**582**	**50.000**	**1.000**
**TRINITY_DN21561_c0_g2_i1_alb**	**Jasmonic acid‐amido synthetase JAR1‐like**	**954**	**50.000**	**1.000**
**TRINITY_DN23226_c0_g1_i4_alb**	**UDP‐galactose/UDP‐glucose transporter 3**	**2402**	**1.238**	**1.000**
**TRINITY_DN25178_c0_g1_i1_alb**	**Inositol phosphorylceramide glucuronosyltransferase 1**	**1712**	**1.128**	**1.000**
TRINITY_DN26945_c0_g1_i1_alb	Threonine dehydratase biosynthetic, chloroplastic	2395	50.000	1.000
TRINITY_DN27700_c3_g4_i1_alb	QWRF motif‐containing protein 3	600	50.000	0.863
TRINITY_DN24641_c0_g1_i4_alb	1,2‐dihydroxy‐3‐keto‐5‐methylthiopentene dioxygenase 2	1170	50.000	0.870
TRINITY_DN27700_c3_g4_i1_alb	QWRF motif‐containing protein 3	600	50.000	0.870
TRINITY_DN33538_c0_g2_i1_alb	Subtilisin‐like protease SBT2.6	587	47.926	0.870
TRINITY_DN27581_c2_g1_i1_alb	Clathrin light‐chain 2‐like	1284	1.046	0.909
TRINITY_DN33021_c0_g1_i1_alb	Uncharacterized protein LOC109706678 isoform X2	272	1.064	0.862
TRINITY_DN10315_c1_g1_i1_alb	NA	693	50.000	1.000
TRINITY_DN17090_c0_g1_i1_alb	NA	579	1.230	1.000
TRINITY_DN28956_c1_g2_i3_alb	NA	480	46.590	1.000
	NA	920	50.000	1.000

Note

Transcripts under positive selection in the Ka/Ks analysis and with *F*_ST_ values between species higher than 0.85. Transcript name, Blast description, transcriptome length, and Ka/Ks ratios of each transcript, and *F*_ST_ of the corresponding RAD loci. In bold are genes under positive selection with putative function in reproductive isolation between *Pitcairnia albiflos* and *Pitcairnia staminea*.

### 3.5 Approximate Bayesian computation

The posterior probabilities from ABC analysis pointed to isolation with decreased migration (Model V; Figure 2) as the most likely scenario of divergence between *P. staminea* and *P. albiflos*. For 5000 retained simulations (5% of the total simulations), the posterior probabilities obtained for this scenario using the neural network algorithm were 0.9029 with PCA transformed data (Figure 2). The cross‐validation tests indicated that isolation with decreased migration model (V) is easily distinguishable from the other scenarios with both datasets (Table S4). All observed summary statistics fitted the range of simulated statistics (Figure S3).

The parameter estimates, based on 5000 posterior samples simulated under scenario V, suggested that the *P. albiflos* population has a three‐time larger effective size than *P. staminea* (Nalb = 16,673; Nsta = 5906; Table 2). Divergence between species was estimated in the Pliocene, 4.7 Mya (CI = 1.3–8.5 Mya). The estimated migration parameters, in turn, showed that recent migration rates were lower in both directions compared to old migration rates (Table 2).

**TABLE 2:** Ranges of priors used to generate the parameters in the msABC simulations for the divergence scenarios between *Pitcairnia albiflos* (alb) and *Pitcairnia staminea* (sta)

Parameter	Prior settings		Parameters estimations based on Scenario V	
	Range	Scenario	Mean	CI
N0	10^4^	All	–	
NE alb	10–10^5^	All	16,673	4981–34,503
NE sta	10–10^5^	All	5906	1634–13,598
Divergence time	0.5–10 Mya	All	4.7 Mya	1.3–8.5 Mya
Old mig alb → sta	10^−3^–1	II, III, and V	0.47	0.04–0.86
Old Tmig alb → sta	0.25–0.5 Mya	III and V	0.95 Mya	0.696–1.210 Mya
Old mig sta → alb	10^−3^–1	II, III. and V	0.5	0.03–0.96
Old Tmig sta → alb	0.25–0.5 Mya	III and V	0.518 Mya	0.350–0.684 Mya
Mig alb → sta	10^−5^–10^−2^ (10^−6^–10^−3^)^a^	II, IV, and V	4 × 10^−4^	9.8 × 10^−6^–8.7 × 10^−4^
Tmig alb → sta	0–0.25 Mya	V	0.016 Mya	0.004–0.025 Mya
Mig sta → alb	10^−5^–10^−2^ (10^−6^–10^−3^)^a^	II, IV, and V	1.5 × 10^−4^	9 × 10^−6^–3 × 10^−4^
Tmig sta → alb	0–0.25 Mya	V	0.247 Mya	0.063–0.466 Mya

Note

Models are described in Figure 2. Model I: isolation with no migration; Model II: isolation with migration; Model III: isolation with old migration; Model IV: secondary contact; Model V: isolation with decreased migration. For all simulations, the parameters were sampled from the uniform distribution within their respective ranges. Posterior parameters estimation with neural network (neuralnet) method from 5000 simulations under best model (scenario V – isolation with decreased migration) in an approximate Bayesian computation framework.

Abbreviations: alb, *P. albiflos* population; CI, confidence interval; Mig, migration rate; N0, ancestral effective population size; NE, effective population size; sta, *P. staminea* population; Tmig, time of migration.

^a^ Prior range applied only for scenario V.

## 4 DISCUSSION

Our results showed high genomic differentiation between the hybridizing species *P. albiflos* and *P. staminea*. In fact, the estimated divergence time occurred during the Pliocene (4.7 Mya ranging from 1.3 to 8.5 Mya), which likely allowed them to accumulate genome‐wide changes. In addition, we detected a total of 17 of 4165 loci under positive selection with high genetic differentiation (*F*_ST_ > 0.85), 12 of them were annotated and four candidate genes were identified to be involved in reproductive isolation. These four candidate genes were previously associated with the function of pollen development, pollen tube germination and orientation, abiotic stress, and flower scent for several other plant species. Finally, our modeling approach revealed divergence with gradual reduction in gene flow between these species over time, strongly suggesting sympatric speciation between these two narrow endemic species.

### 4.1 Evidence of genome‐wide changes

The process of speciation comprises of the gradual accumulation of differences between populations in small steps (Coyne et al., 2004; Darwin, 1859; Seehausen et al., 2014) and it can involve genetically localized or genome‐wide changes (Nosil et al., 2017). Our study revealed genome‐wide differentiation between *P. staminea* and *P. albiflos* (Figure S1), as indicated by the high number of private alleles, high and significant genetic differentiation (*F*_ST_) (with and without detected outlier loci), and the presence of two distinct groups with significant differences in allele frequencies between them. Our results are in agreement with those previously found for this hybrid zone of *P. staminea* and *P. albiflos* by Palma‐Silva et al. (2011, 2015) using a limited number of microsatellites loci. These results are also in agreement with Flaxman et al. (2014), who demonstrated with models of divergence with weak selection and strong gene flow that linkage disequilibrium with divergent selection forces can jointly reach threshold levels in the transition point and enhance a positive feedback process, causing genome‐wide changes. Thus, effects of reproductive isolation eventually evolve to the whole genome (Nosil et al., 2017).

The parameter estimates under the scenario of divergence with decreased migration (ABC analysis – Model V) (Figure 2) suggested that *P. staminea* and *P. albiflos* have diverged approximately 4.7 Mya (CI = 1.3–8.5 Mya) during the Pliocene. Such ancient divergence might have enabled the accumulation of the genome‐wide differences detected by our analyses, probably due to the accumulation of reproductive barriers and decreasing levels of interspecific gene flow, as expected in the speciation continuum (Seehausen et al., 2014; Stankowski & Ravinet, 2021). Moreover, the divergence time during the Pliocene also suggests that speciation history between *P. albiflos* and *P. staminea* is possibly connected to the neotectonic processes, since Southeastern Brazil experienced the third tectonic pulse probably during Pliocene, which led to the modern topography of Serra do Mar region (Almeida, 1976). Further analyses are necessary for robust demographic estimation, which may help us in future studies to understand the main drivers of the split of ancestral population and to guide us for which “speciation genes” to look for.

### 4.2 Genomic regions possibly associated with reproductive isolation

Regions of high genomic differentiation are often assumed to have arisen due to reproductive barriers, while homogenizing gene flow decreases differentiation elsewhere in the genome (Ravinet et al., 2017). Therefore, several genome scan studies have used next‐generation sequencing to map candidate genomic regions underlying reproductive isolation, namely candidate barrier loci (reviewed by Ravinet et al., 2017). Our analysis of Ka/Ks ratio from de novo assembled and annotated transcriptomes and the genetic differentiation index of corresponding RAD sequences revealed 17 putative regions under positive selection (Ka/Ks > 1) with high genetic differentiation (*F*_ST_ > 0.85) between species. Of these 17 transcripts, 12 were previously annotated with candidate functions identified (see Table 1). From these annotated regions, we discuss below four putative genes that might be associated with reproductive isolation between *P. staminea* and *P. albiflos*.

One of these candidate genes retrieved by our analysis is the “major pollen allergen,” which was identified to have an important role in pollen tube wall reformation during germination of olives pollen (Barral et al., 2020). The UDP‐galactose/UDP‐glucose transporter 3 (GO: 0072334), in turn, has been demonstrated to carry sugars necessary for the pollen germination and pollen tube growth in *Arabidopsis* (Decker et al., 2017). The third gene likely under positive selection, and also an outlier detected by PCAdapt analysis, is the inositol phosphorylceramide glucuronosyltransferase 1 (GO: 1990482). A study done with mutants for these genes in *Arabidopsis* showed that it affects the orientation of the pollen tube growth (Tartaglio et al., 2017). It is possible that the functions of these three candidate genes have an important role in pollen–pistil interactions and incompatibility or incongruence occur due to genetic divergences between the two species (Hiscock & Allen, 2008). In fact, pollen germination and pollen tube growth are limiting factors in plant reproduction (Dafni & Firmage, 2000). Moreover, pollen–pistil incompatibility has been shown to act as postpollination barrier in other closely related plant species occurring in sympatry (e.g., *Opuntia elata* and *O. retorsa*: Fachardo & Sigrist, 2020; *Streptanthus breweri* and *S. hesperidis*: Christie & Strauss, 2019). Therefore, pollen–pistil incompatibility as reproductive isolation barrier was suggested to be responsible in maintaining species boundaries in assemblages of sympatric bromeliad species (Cavalcante et al., 2020; Matallana et al., 2016; Ramírez‐Rosas et al., 2020).

The fourth gene putatively under positive selection and with high genetic differentiation between the species (Table 1) is the jasmonic acid‐amido synthetase (GO: 0009864), which was previously shown to be involved in pollen development as well as in response to abiotic stress (Turner et al., 2002) and in flower scent biosynthesis (Xu et al., 2019). The jasmonic acid‐amido synthetase, in particular, may also play a role in prepollination isolation, since it is also involved in flower scent biosynthesis that likely affects pollinators’ attraction. Such floral trait often differs between closely related species, suggesting that pollinator behavior mediated by sensory traits can be a key factor for the establishment of reproductive isolation during the early phase of species divergence in angiosperms (Hopkins, 2013; Schiestl & Schluter, 2009). Accordingly, Wendt et al. (2001) observed that *P. staminea* and *P. albiflos* diverge in the production of floral scent as well as in the color of the flowers and, as a consequence, in their effective pollinators. *P. albiflos* have white and scented flowers and are nocturnally pollinated by bats and hawk moths and *P. staminea* have red and scentless flowers and are diurnally pollinated by butterflies. Therefore, it is possible that jasmonic acid‐amido evolved together with other genes that determine such flower traits divergence between the two species. In fact, floral isolation can be an important process during plant speciation by establishing reproductive isolation and facilitating the accumulation of genetic differences and thus the evolution of genetic barriers (Rieseberg & Willis, 2007).

The empirical evidence for positive selection on loci underlying genetic incompatibilities indicates that genomic conflict may be a common mechanism driving the speciation process (Seehausen et al., 2014). Indeed, our results suggest that candidate barrier genes identified here may be involved in pre‐ and postpollination prezygotic barriers due to their function in pollen development, pollen tube germination and orientation, and floral scent biosynthesis. Understanding how specific barriers contribute to reproductive isolation offers insight into the initial forces driving divergence and the evolutionary and ecological processes responsible for maintaining diversity (Coyne et al., 2004). Therefore, future studies with other sympatric populations of these two species applying the whole‐genome resequencing approach might be able to detect patterns in gene exchange and gene differentiation between them, elucidating questions about the evolution of reproductive barriers and deepening our comprehension of the importance of structural variations (i.e., deletions, duplications, inversions, and translocations), helping us to disentangle molecular basis and genomic architecture of reproductive isolation across the speciation continuum.

### 4.3 Speciation with gradual migration reduction

Understanding patterns of interspecific gene flow over time is fundamental for studying the genomic basis of reproductive isolation. Furthermore, testing and quantifying the extent of gene flow is a crucial prerequisite for interpreting genomic analyses of hybrid zones correctly (Ravinet et al., 2017). Our results from the ABC analysis indicated the model of speciation with decreased gene flow between *P. staminea* and *P. albiflos* over time as the best one (higher posterior probabilities). This result provides valuable support of sympatric speciation between these two narrow endemic species. In agreement, the two models that do not include gene flow had an extremely low posterior probability (0.0526 and 0.0000), strongly rejecting allopatric speciation with or without secondary contact (Models IV and I, respectively; Figure 2). The ABC framework has been widely used in speciation research, and other studies have also described scenarios of divergence with gene flow in other organisms (e.g., amphibians: Bessa‐Silva et al., 2020; birds: Wang et al., 2021; and plants: Muniz et al., 2022). Indeed, the prevalence of gene flow between diverging entities has been demonstrated by Roux et al. (2016) using genomic data from 61 independent speciation events spanning a broad range of the animal kingdom under an ABC framework, confirming the high incidence of semi‐isolated species in nature. When speciation occurs in the absence of geographic barriers, as our results suggest for the studied *Pitcairnia* species, there is a powerful demonstration that divergent selection can overcome the homogenizing effects of gene flow and recombination and, thus, lead species to diverge (Coyne et al., 2004; Ravinet et al., 2017).

Additionally, the parameter estimates derived from the ABC showed low rates of current migration (~10^−4^ for both directions), which may have allowed the species to maintain the genome‐wide differences accumulated over time (Figure S1). Also, the higher past migration rates detected here (~0.5 for both directions) possibly indicate an increase in the reproductive isolation since divergence. Two other studies on plants (i.e., *Senecio*: Filatov et al., 2016, *Howe*: Papadopulos et al., 2019) also demonstrated through demographic analysis a scenario of speciation under gene flow with decreased migration since divergence, confirming much lower migration rate in the present compared to the past. Given that divergent selection might act in contrasting directions in two populations, it can also produce genetic differentiation by promoting reproductive isolation barriers to gene flow (Nosil et al., 2009). Accordingly, previous studies on these sympatric *Pitcairnia* species evidenced strong prezygotic (Palma‐Silva et al., 2011, 2015; Wendt et al., 2001) and postzygotic (Palma‐Silva et al., 2015) barriers acting in this hybrid zone despite the presence of hybrids (Palma‐Silva et al., 2011, 2015; Wendt et al., 2001) and backcrosses (Palma‐Silva et al., 2011). Although these scenarios and identified putative candidate genes associated with reproductive isolation between *P. staminea* and *P. albiflos* need to be investigated more thoroughly, the pattern observed here suggest that divergent selection may play an important role on the speciation process of *P. staminea* and *P. albiflos*.

Studies that suggest speciation with gene flow have been demonstrated for organisms of Neotropical region, including animals (e.g., *Heliconius* butterflies: Martin et al., 2013; *Cnemidophourus* lizards: Oliveira et al., 2015; *Amphilophus* cichlid fishes: Kautt et al., 2016, 2020; and treefrog *Boana raniceps*: Camurugi et al., 2021) and plants (e.g., magic flowers *Achimenes*: Roberts & Roalson, 2018; and *Plathymenia reticulata*: Muniz et al., 2022). In fact, interspecific gene flow can contribute to the pool of standing genetic variation and thus to diversification of species (Brawand et al., 2015; Seehausen, 2004) and showed to have an important role in diversification and adaptive radiation of bromeliads (Lexer et al., 2016; Loiseau et al., 2021; Palma‐Silva, Ferro, et al., 2016; Palma‐Silva, Leal, et al., 2016). More specifically, species of the genus *Pitcairnia* have shown a strong tendency to remain genome porous for extended periods of time (Mota et al., 2019, 2020; Palma‐Silva et al., 2011). Thus, our study helps to understand the patterns of sympatric speciation and contributes to unravel the role of interspecific gene flow to the divergence process between sympatric species of Neotropical adaptive radiation groups.

## CONFLICT OF INTEREST

The authors declare no conflicts of interests in this study.

## AUTHOR CONTRIBUTIONS

**Marilia Manuppella Tavares:**Conceptualization (equal); Data curation (lead); Formal analysis (lead); Writing – original draft (lead); Writing – review & editing (lead). **Milene Ferro:** Conceptualization (equal); Data curation (equal); Formal analysis (equal); Writing – original draft (equal); Writing – review & editing (equal). **Bárbara Leal:** Conceptualization (equal); Formal analysis (equal); Supervision (equal); Writing – original draft (equal); Writing – review & editing (equal). **Clarisse Palma‐Silva:** Conceptualization (lead); Funding acquisition (lead); Project administration (lead); Supervision (lead); Writing – original draft (equal); Writing – review & editing (equal).

## Supporting information

Fig S1

Fig S2

Fig S3

Table S1‐S4

Appendix S1

Appendix S2

Appendix S3

## Data Availability

The raw sequence data of each transcriptome library are freely available from NCBI short read archives and deposited in GenBank SRA under the Accession no. SRP064189. Raw RAD‐seq data have been deposited in the NCBI SRA database (BioProject ID: PRJNA807675; BioSample accessions: SAMN25995380–SAMN25995403). The Ipyrad outputs generated from de novo assembly of RAD‐seq data and final de novo transcriptome assembly per species uploaded are available on the Dryad Digital Repository https://doi.org/10.5061/dryad.qjq2bvqhw.
