# Supplementary material for: Speciation with gene flow between two Neotropical sympatric species (Pitcairnia spp.: Bromeliaceae)
Source: Ecol Evol. 2022 Apr 29;12(5):e8834. doi: 10.1002/ece3.8834 (PMC9055293; doi:10.1002/ece3.8834)
Supplement: Supplementary file 1 — Fig S1 [file ECE3-12-e8834-s001.pptx]

## Slide 1
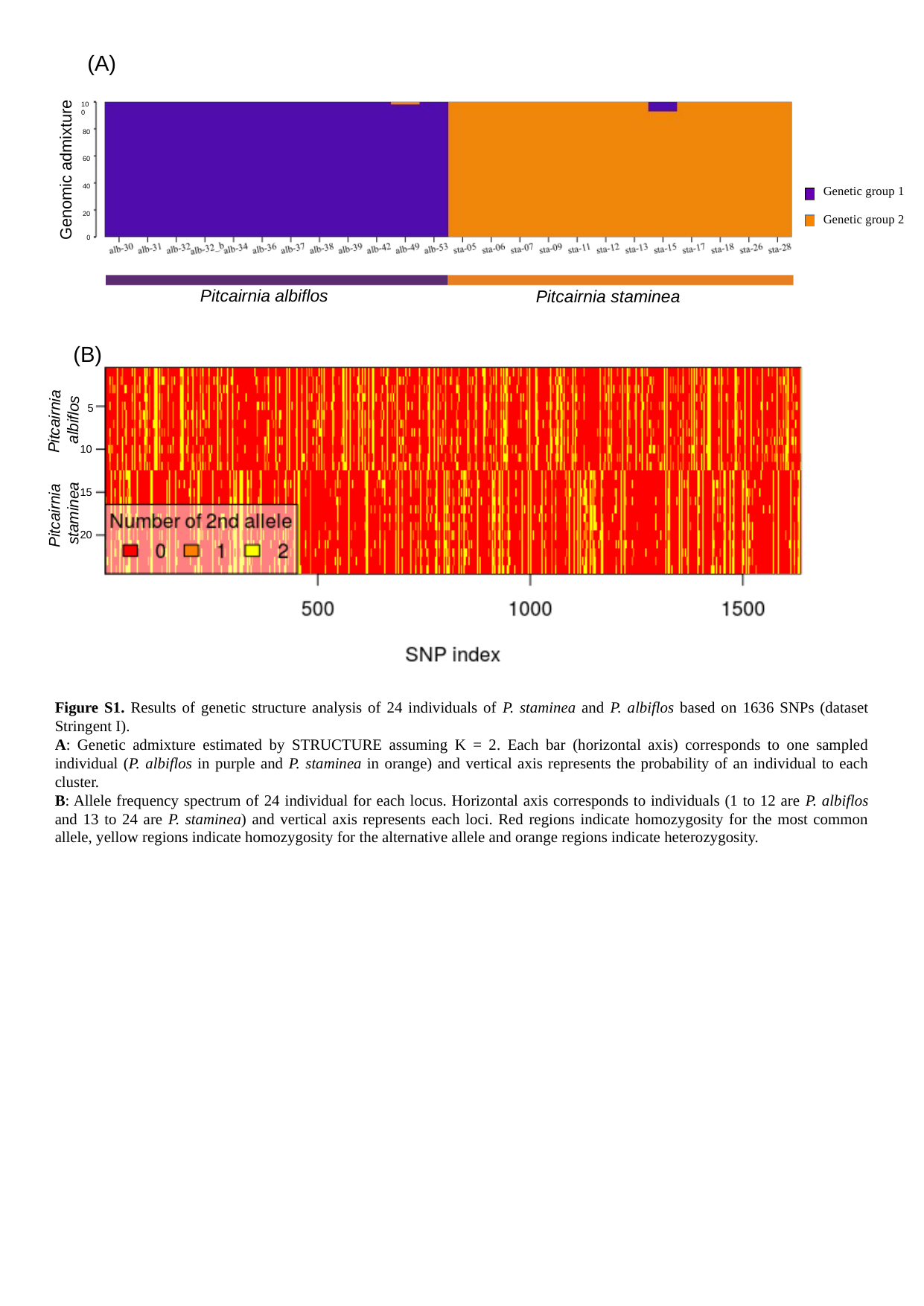

(A)
100
80
60
40
20
0
Genomic admixture
Genetic group 1
Genetic group 2
Pitcairnia albiflos
Pitcairnia staminea
(B)
5
Pitcairnia albiflos
10
15
Pitcairnia staminea
20
Figure S1. Results of genetic structure analysis of 24 individuals of P. staminea and P. albiflos based on 1636 SNPs (dataset Stringent I).
A: Genetic admixture estimated by STRUCTURE assuming K = 2. Each bar (horizontal axis) corresponds to one sampled individual (P. albiflos in purple and P. staminea in orange) and vertical axis represents the probability of an individual to each cluster.
B: Allele frequency spectrum of 24 individual for each locus. Horizontal axis corresponds to individuals (1 to 12 are P. albiflos and 13 to 24 are P. staminea) and vertical axis represents each loci. Red regions indicate homozygosity for the most common allele, yellow regions indicate homozygosity for the alternative allele and orange regions indicate heterozygosity.
