# Supplementary material for: Speciation with gene flow between two Neotropical sympatric species (Pitcairnia spp.: Bromeliaceae)
Source: Ecol Evol. 2022 Apr 29;12(5):e8834. doi: 10.1002/ece3.8834 (PMC9055293; doi:10.1002/ece3.8834)
Supplement: Supplementary file 2 — Fig S2 [file ECE3-12-e8834-s004.pptx]

## Slide 1
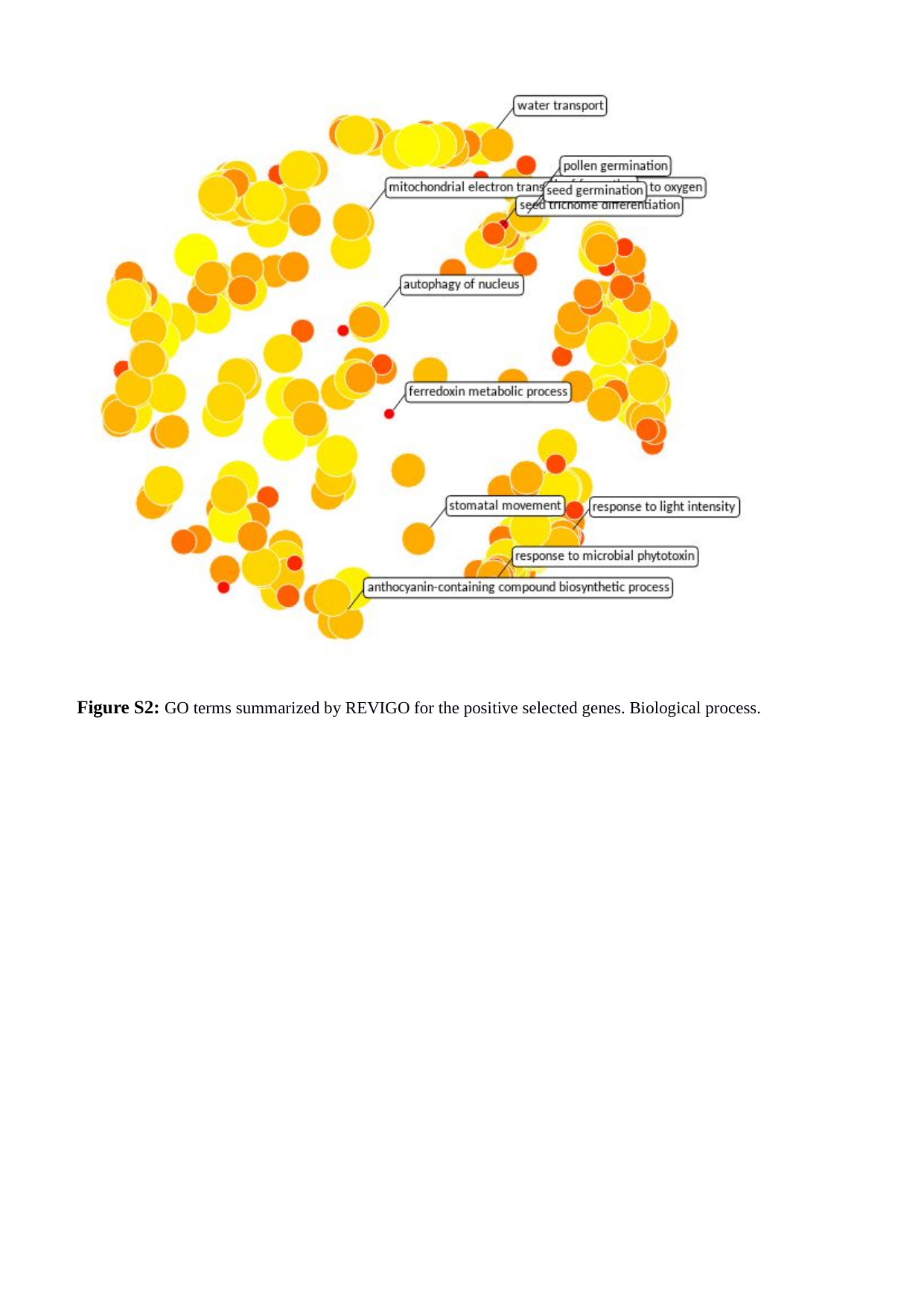

Figure S2: GO terms summarized by REVIGO for the positive selected genes. Biological process.
