# Supplementary material for: Speciation with gene flow between two Neotropical sympatric species (Pitcairnia spp.: Bromeliaceae)
Source: Ecol Evol. 2022 Apr 29;12(5):e8834. doi: 10.1002/ece3.8834 (PMC9055293; doi:10.1002/ece3.8834)
Supplement: Supplementary file 3 — Fig S3 [file ECE3-12-e8834-s002.pptx]

## Slide 1
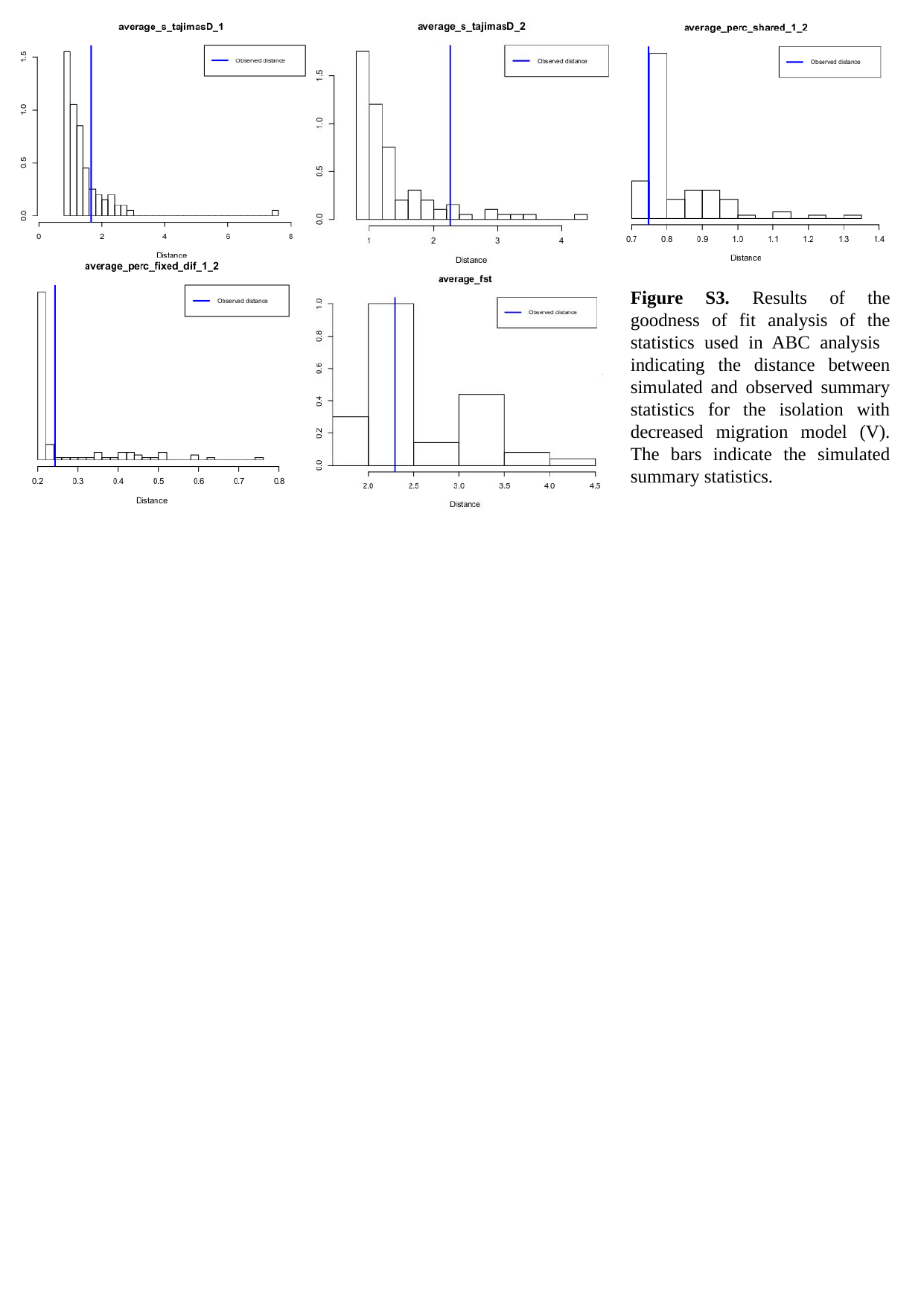

Figure S3. Results of the goodness of fit analysis of the statistics used in ABC analysis indicating the distance between simulated and observed summary statistics for the isolation with decreased migration model (V). The bars indicate the simulated summary statistics.
