# Supplementary material for: Speciation with gene flow between two Neotropical sympatric species (Pitcairnia spp.: Bromeliaceae)
Source: Ecol Evol. 2022 Apr 29;12(5):e8834. doi: 10.1002/ece3.8834 (PMC9055293; doi:10.1002/ece3.8834)
Supplement: Supplementary file 4 — Table S1‐S4 [file ECE3-12-e8834-s006.pdf]

SUPPLEMENTARY MATERIAL

TITLE: Speciation with gene flow between two Neotropical sympatric species (*Pitcairnia spp:* Bromeliaceae)

Marília Manuppella Tavares[1#], Milene Ferro[2#], Bárbara Simões Santos Leal[1] and Clarisse Palma-Silva[1]*

[1]Departamento de Biologia Vegetal, Instituto de Biologia, Universidade Estadual de Campinas, Campinas, SP 13083-862, Brazil

[2]Departamento de Biologia Geral e Aplicada, Universidade Estadual Paulista, Rio Claro, SP.

# These authors contributed equally to this work

* Corresponding to:

Dra. Clarisse Palma da Silva

Laboratório de Ecologia Evolutiva e Genômica de Plantas

Programa de PG em Biologia Vegetal

Departamento de Biologia Vegetal

Universidade Estadual de Campinas, UNICAMP

Campinas, SP, Brasil

13083-862

Email: cpalma@unicamp.br

Table S1. Number of reads sequenced per individual of *P. albiflos* and *P. staminea* (Bromeliaceae); number of reads with phred score > 20; GC content and contig length.

**Individual**	**reads raw**	**reads passed filter (Q>20)**	**GC content**	**Contig length (pb)**
alb-30_1	3802648	3802027	0.5255	91
alb-31_1	3905896	3905207	0.5240	91
alb-32_1	3835133	3834498	0.5274	91
alb-32b_1	3385144	3384549	0.5321	91
alb-34_1	3932337	3931710	0.5282	91
alb-36_1	2138001	2137659	0.5351	91
alb-37_1	2350634	2350208	0.5340	91
alb-38_1	2141677	2141297	0.5426	91
alb-39_1	2475184	2474783	0.5357	91
alb-42_1	3483270	3482712	0.5332	91
alb-49_1	3932303	3931588	0.5686	91
alb-53_1	3494892	3494269	0.5086	91
sta-05_1	3982486	3981744	0.5250	91
sta-06_1	3912463	3911773	0.5066	91
sta-07_1	3328042	3327485	0.5073	91
sta-09_1	2621792	2621319	0.5013	91
sta-11_1	2200939	2200589	0.5058	91
sta-12_1	3073199	3072630	0.5285	91
sta-13_1	2919301	2918742	0.5108	91
sta-15_1	3725544	3724935	0.5303	91
sta-17_1	3713208	3712569	0.5367	91
sta-18_1	3025808	3025291	0.5071	91
sta-26_1	4004967	4004248	0.5372	91
sta-28_1	3984575	3983908	0.5344	91

TableS2. Summary of preprocessing reads and *de novo* assembled transcriptomes for *Pitcairnia albiflos and Pitcairnia staminea* and their tissues (flower and leaf).

**Analysis steps**	***Pitcairnia albiflos***		***Pitcairnia staminea***	
	**Flower**	**Leaf**	**Flower**	**Leaf**
Total reads count	109,789,092	113,952,806	113,952,806	103,580,018
Clean reads SeqyClean	105,648,118	109,351,882	93,440,096	99,856,242
Total assembled bases	149,230,092		152,638,050	
Total Trinity transcripts	147,097		152,819	
CDS	145,788		151,643	
Percent GC (%)	44.99		45.24	
Total Trinity genes	93,806		102,142	
N50 all transcripts	1799		1816	
Average contig (bp)	1014.50		998.82	

Table S3. Commands used to produce the simulations under the five scenarios of divergence with the msABC. locfile.txt corresponds to the file containing information about sample size of each locus, length (pb) of each locus and the mutation rate.

Divergence model	msABC command line
**Model I**	msABC 24 NUMSIMS --dur-mode -I 2 12 12 -en 0 1 -U 0.001 10 -en 0 2 -U 0.001 10 -ej -U 2.5 50 2 1 --frag-begin --finp locfile.txt --N 10000 --frag-end --verbose
**Model II**	msABC 24 NUMSIMS --dur-mode -I 2 12 12 -m 1 2 -U 0.4 400 -m 2 1 -U 0.4 400 -en 0 1 -U 0.001 10 -en 0 2 -U 0.001 10 -ej -U 2.5 50 2 1 --frag-begin --finp locfile.txt --N 10000 --frag-end --verbose
**Model III**	./msABC 24 NUMSIMS --dur-mode -I 2 12 12 -em -U 0 1.25 2 1 0 -em -U 0 1.25 1 2 0 -en 0 1 -U 0.001 10 -en 0 2 -U 0.001 10 -em -U 1.25 2.5 1 2 -U 0.4 400 -em -U 1.25 2.5 2 1 -U 0.4 400 -ej -U 2.5 50 2 1 --frag-begin --finp locfile.txt --N 10000 --frag-end --verbose
**Model IV**	./msABC 24 NUMSIMS --dur-mode -I 2 12 12 -en 0 1 -U 0.001 10 -en 0 2 -U 0.001 10 -m 2 1 -U 0.4 400 -m 1 2 -U 0.4 400 -em -U 1.25 2.5 1 2 0 -em -U 1.25 2.5 2 1 0 -ej -U 2.5 50 2 1 --frag-begin --finp locfile.txt --N 10000 --frag-end --verbose
**Model V**	msABC 24 NUMSIMS --dur-mode -I 2 12 12 -en 0 1 -U 0.001 10 -en 0 2 -U 0.001 10 -m 2 1 -U 0.04 40 -m 1 2 -U 0.04 40 -em -U 1.25 2.5 1 2 -U 40 40000 -em -U 1.25 2.5 2 1 -U 40 40000 -ej -U 2.5 50 2 1 --frag-begin --finp locfile.txt --N 10000 --frag-end --verbose

Table S4. Confusion matrix results using neural network (neuralnet) method based on cross-validation test using 100 simulated samples for each model with statistics transformed by Principal Component Analysis.

Simulated scenarios	Estimated scenarios					Total
	I	II	III	IV	V	
**I**	81	0	13	0	6	100
**II**	0	77	0	23	0	100
**III**	28	0	71	0	1	100
**IV**	0	49	0	51	0	100
**V**	3	8	1	2	86	100
